# Diverse Misfolded Conformational Strains and Cross-seeding of Misfolded Proteins Implicated in Neurodegenerative Diseases

**DOI:** 10.3389/fnmol.2019.00158

**Published:** 2019-07-09

**Authors:** Kwang Hun Lim

**Affiliations:** Department of Chemistry, East Carolina University, Greenville, NC, United States

**Keywords:** misfolding, prion, cross-seeding, α-synuclein, tau, conformational strain

## Abstract

Numerous neurodegenerative diseases including prion, Alzheimer’s and Parkinson’s diseases are characterized by accumulation of protein aggregates in brain. Prion disease is unique in that the natively folded prion protein forms diverse misfolded aggregates with distinct molecular conformations (strains), which underlie different disease phenotypes. In addition, the conformational strains are able to self-propagate their unique conformations by recruiting normal protein monomers and converting their conformations to misfolded conformers. There is an increasing body of evidence that suggests other aggregation-prone proteins including tau and α-synuclein associated with Alzheimer’s and Parkinson’s diseases, respectively, also behave like a prion that has conformational strains with self-propagation (seeding) property. Moreover, misfolded protein aggregates can promote misfolding and aggregation of different proteins through cross-seeding, which might be associated with co-occurrence of multiple neurodegenerative diseases in the same patient. Elucidation of diverse conformational strains with self-propagation capability and of molecular basis for the cross-talk between misfolded proteins is essential to the development of effective therapeutic intervention.

## Introduction

The hallmark of numerous neurodegenerative diseases is extra- or intra-cellular deposits of misfolded protein aggregates in the central nervous systems (CNS; Sacchettini and Kelly, 2002; Jahn and Radford, 2008; Eisenberg and Jucker, 2012; Knowles et al., 2014; Chiti and Dobson, 2017). Protein misfolding and aggregation involves conformational changes from native polypeptides to aggregation-prone misfolded conformers, which self-assemble into fibrillar aggregates. The fibrillar protein assemblies adopt cross-β structures in which β-strands are arranged in an orientation perpendicular to the aggregation axis (Tycko, 2006; Riek and Eisenberg, 2016). More than 20 polypeptides have been identified to undergo misfolding transition associated with diverse human disorders including Alzheimer’s and Parkinson’s diseases, amyloidosis, type 2 diabetes, and prion diseases (Campioni et al., 2010; Eisenberg and Jucker, 2012; Chiti and Dobson, 2017).

The unique characteristics of prion protein is the ability to form diverse molecular conformations (strains) associated with different disease phenotypes (Collinge, 2010; Frost and Diamond, 2010; Westermark and Westermark, 2010; Jucker and Walker, 2013). Misfolded prion aggregates are also capable of propagating their unique conformations and spreading pathological conditions between cells and tissues. Growing evidence suggests that other aggregation-prone proteins have prion-like properties of conformational strains associated with distinct disease phenotypes and self-propagation of the conformations through seeding (Frost and Diamond, 2010; Kim and Holtzman, 2010; Lee et al., 2010; Westermark and Westermark, 2010; Gerson et al., 2016; Goedert et al., 2017). Recent high-resolution structural studies using solid-state NMR and cryo-EM has begun to reveal near-atomic resolution structures of several filamentous aggregates (Colvin et al., 2016; Tuttle et al., 2016; Fitzpatrick et al., 2017; Falcon et al., 2018; Guerrero-Ferreira et al., 2018; Li B. et al., 2018). The high-resolution structures showed that a single polypeptide can adopt distinct filamentous aggregates (strains) with different molecular structures. In addition, misfolded strains with different molecular structures induce distinct disease phenotypes in animal models (Peelaerts et al., 2015), supporting the prion hypothesis for the neurodegenerative diseases.

Traditionally, misfolding and aggregation of a single protein was believed to be involved in each pathological process. For example, intracellular deposition of misfolded α-synuclein is linked to Parkinson’s disease (PD), while intraneuronal accumulation of filamentous tau is associated with Alzheimer’s diseases (AD). However, there is mounting evidence that suggests AD and PD pathologies are significantly overlapped presumably due to synergistic interactions between tau and α-synuclein, highlighting the complexity of Alzheimer’s diseases and related dementia (ADRD) pathogenesis (Clinton et al., 2010; Moussaud et al., 2014; Castillo-Carranza et al., 2018). Understanding molecular basis for the misfolded conformational strains with self-propagation properties and cross-seeding between the misfolded proteins would, therefore, be crucial to the development of therapeutic interventions for the debilitating human disorders.

## Misfolded Conformational Strains

**Figure 1:**
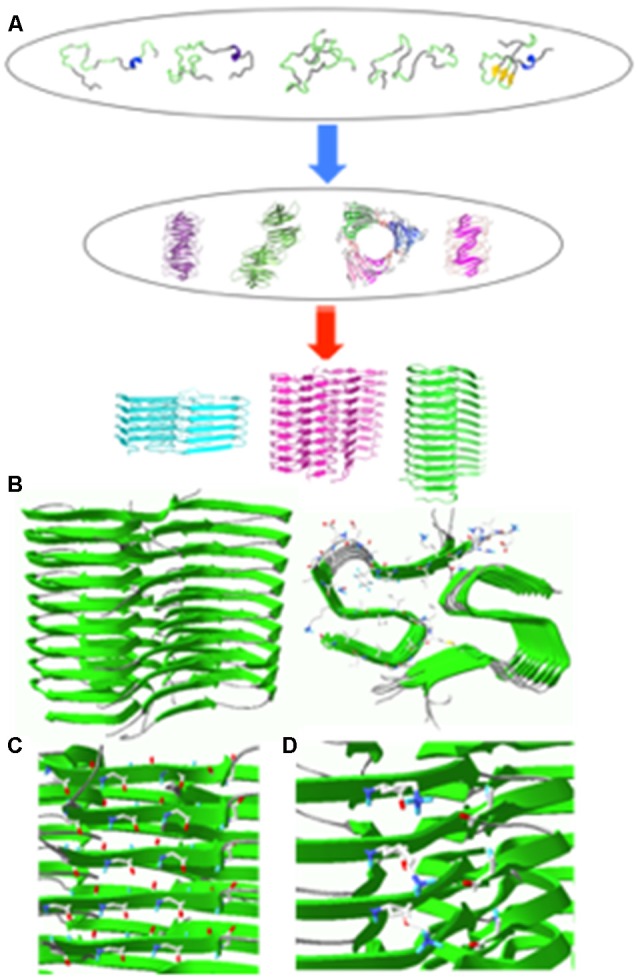
**(A)** Schematic diagram of aggregation process for an intrinsically disordered protein. Some of the aggregation-prone conformers in the conformational ensemble may form various oligomers that can self-assemble into fibrillar aggregates with different molecular conformations depending on different environments (Jahn and Radford, 2005). **(B)** Solid-state NMR structure of Aβ(1–42) filaments (sideview, left; top view, right). **(C)** Hydrogen bonding interactions between carbonyl carbon (red) and amide hydrogen (blue). **(D)** Salt bridges between NH3+ of K28 (blue) and COO^−^ of A42 (red) in Aβ(1–42) filaments. The filament structures were drawn with PDB accession number (5KK3).

Main structural feature of misfolded protein aggregates is the cross-β fold in which β-strands are stacked in an orientation perpendicular to the aggregation axis (bottom in Figure 1A). Major driving force for the formation of cross-β structure is the hydrogen bonding interactions between the main chain carbonyl oxygen and amide hydrogen (Sawaya et al., 2007; Lu et al., 2013; Riek and Eisenberg, 2016). Misfolded filamentous aggregates share the common structural motif irrespective of amino acid sequence. However, recent high-resolution structural studies revealed that misfolded filamentous aggregates can adopt diverse molecular structures within the common cross-β fold (Eichner and Radford, 2011; Tycko, 2015; Annamalai et al., 2016; Iadanza et al., 2018).

A majority of aggregation-prone proteins are intrinsically disordered under the physiological condition. These proteins include β-amyloid (Aβ) peptides, α-synuclein, and tau associated with various age-related neurodegenerative diseases. Intrinsically disordered proteins adopt a heterogeneous ensemble of conformations. The diverse conformers in the conformational ensemble might be induced to form distinct misfolded aggregates (strains) with different molecular conformations depending on experimental conditions (Figure 1A). Indeed various pathological proteins including Aβ peptides, α-synuclein and tau were shown to be able to adopt distinct strains with different molecular structures, which might be linked to phenotype diversities of the neurodegenerative diseases (Guo et al., 2013; Riek and Eisenberg, 2016; Goedert et al., 2017; Falcon et al., 2018; Peng et al., 2018a,b).

It is, therefore, of critical importance to characterize various strains with diverse molecular conformations to understand the molecular basis for the prion-like propagation and spreading of pathological protein aggregates. In this review article, diverse high-resolution structures of filamentous aggregates formed by three aggregation-prone proteins (Aβ peptides, α-synuclein, and tau) will be discussed.

### Aβ Peptides

Aβ peptides are 36–43 residue peptides derived by the cleavage of amyloid precursor protein. Major components of the amyloid plaques observed in Alzheimer’s patients are Aβ(1–40) and Aβ(1–42) peptides. Over the last two decades, structural studies have been predominantly focused on misfolded aggregates of the two Aβ peptides. The first structural model for Aβ(1–40) fibrillar aggregates was reported on the basis of solid-state NMR constraints (Petkova et al., 2002; Tycko, 2011; Lu et al., 2013). Later, higher resolution structures were determined by solid-state NMR and cryo-EM for the Aβ(1–42) fibrillar aggregates (Lu et al., 2013; Colvin et al., 2016; Wälti et al., 2016; Gremer et al., 2017).

The atomic resolution structure of Aβ(1–42) fibrillar aggregates revealed that the cross-β fold is stabilized by various interactions (Figure 1B). First, the β-strands stacked along the aggregation axis are held together by extensive hydrogen bonding interactions between amide hydrogens and carbonyl carbons in the peptide backbones (Figure 1C, left). Second, hydrophobic interactions between bulky hydrophobic sidechains play an important role in stabilizing the cross-β fold (Figure 1B, right). A variety of hydrophobic interactions through sidechain interdigitations (steric zipper) have been observed in the fibrillar aggregates. Third, salt bridges between acidic and basic side chains help maintain fibrillar structures (Figure 1C, right).

Polymorphic nature of fibrillar aggregates has been also observed for Aβ peptides incubated under different experimental conditions (Tycko, 2015). Two distinct Aβ(1–40) fibrillar aggregates were formed when the samples were incubated with gentle circular agitation and quiescent (unstirred) conditions (Petkova et al., 2005). The agitated fibrils exhibited straight ribbon-type morphologies where protofilaments are laterally associated. On the contrary, quiescent fibrils adopt twisted morphologies in which protofilaments are twisted around each other. Intriguingly, Aβ aggregates seeded by brain extracts from different AD patients were also shown to exhibit distinct molecular structures (Tycko, 2015; Qiang et al., 2017). These experimental results suggest that distinct misfolded strains might be associated with different disease phenotypes. Further investigation of molecular structures of diverse misfolded Aβ aggregates and their biological activities will help understand structure-function relationship of misfolded Aβ aggregates.

### α-Synuclein

α-synuclein is a 140-residue intrinsically disordered protein that is primarily localized at presynaptic terminals of the CNS. Intracellular inclusions containing misfolded α-synuclein aggregates are a hallmark of several neurodegenerative diseases such as PD and dementia with Lewy bodies, collectively termed synucleinopathies (Goedert, 2001; Lashuel et al., 2013).

The presynaptic protein consists of three main regions: the amphipathic N-terminal (1–64), hydrophobic non-Aβ-component (NAC; 65–95), and acidic C-terminal region (96–140). The hydrophobic NAC region that plays a central role in α-synuclein aggregation was shown to be protected by long-range interactions between the N- and C-terminal regions in the natively disordered state of α-synuclein (Bertoncini et al., 2005b; Dedmon et al., 2005). Thus, perturbations of the long-range interactions by single-point mutations and interactions with small molecules led to the formation of filamentous α-synuclein aggregates (Bertoncini et al., 2005a).

Recent structural studies of misfolded α-synuclein aggregates revealed that filamentous α-synuclein aggregates also have different molecular structures depending on the experimental conditions such as pH and salt concentration. Under low salt conditions, α-synuclein filaments have ribbon-type morphology, while twisted morphology was observed at high salt conditions (Bousset et al., 2013). The two distinct filaments were also shown to exhibit distinct disease phenotypes in mice models (Peelaerts et al., 2015).

**Figure 2:**
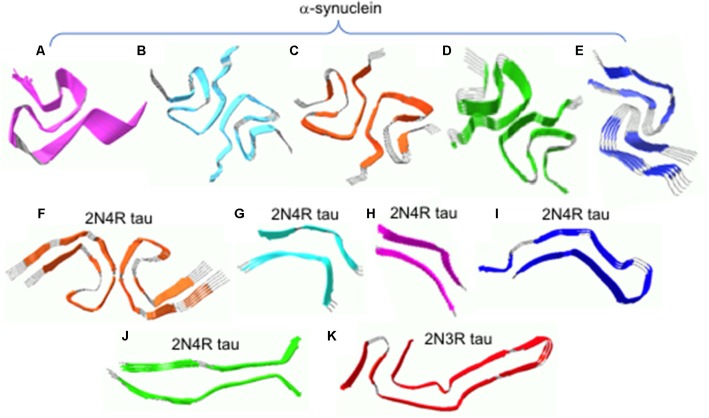
**(A)** Solid-state NMR structure of α-synuclein filaments. **(B–E)** Cryo-EM structures of α-synuclein filaments. **(F)** Cryo-EM structures of tau filaments extracted from an AD patient. **(G–J)** Cryo-EM structures of tau filaments induced by heparin. **(K)** Cryo-EM structures of tau filaments extracted from a PiD patient. The filament structures were drawn with PDB accession numbers, 2N0A **(A)**, 6A6B **(B)**, 6FLT **(C)**, 6CU7 **(D)**, 6CU8 **(E)**, 5O3L **(F)**, 3QJM **(G)**, 3QJP **(H)**, 3QJH **(I)**, 3QJQ **(J)**, and 6GX5 **(K)**.

Very recently, high-resolution structures were determined by solid-state NMR and cryo-EM (Figures 2A–E; Tuttle et al., 2016; Guerrero-Ferreira et al., 2018; Li B. et al., 2018; Li Y. et al., 2018). Solid-state NMR structure of α-synuclein filaments revealed that the filament core consisting of residues 38–97 adopts a Greek-key topology (Figure 2A; Tuttle et al., 2016). Near-atomic high-resolution structures were also solved by cryo-EM at resolution of 3–4 Å (Figures 2B–E). Two cryo-EM structures exhibit a Greek-key type fold similar to that in solid-state NMR structure (Figures 2B,C; Guerrero-Ferreira et al., 2018; Li B. et al., 2018). However, detailed molecular structures such as the location of the turn and sidechain orientations differ in the structures. For example, residues 51–67 are disordered in the solid-state NMR structure, while those regions are well defined in the cryo-EM structure. The sidechain of residue A53 points into the opposite directions. In addition, distinct cross-β folds were observed in α-synuclein filaments formed in the presence of tetrabutylphosphonium bromide (15 mM; Figures 2D,F; Li B. et al., 2018). In particular, the bent b-arch fold in Figure 2E has distinct inter facial regions (residues 46–56), compared to that (residues 68–78) in Figure 2D. The different molecular structures also led to the formation of distinct morphologies, rod and twisted polymorphsin Figures 2D,E, respectively (Li Y. et al., 2018). Filamentous aggregates with different molecular structures and morphologies (surface structure) may interact with the monomers in a different way, leading to differential (cross-) seeding activities. Additional structural studies of the filamentous aggregates and elucidation of their (cross-) seeding activities are, therefore, required to better understand the differential functional properties of the diverse α-synuclein filaments.

### Tau

Tau is an intrinsically disordered 352–441 residue microtubule-binding protein that is abundantly present in the CNS. An alternative splicing of the tau gene leads to six isoforms of the tau protein. The six isoforms consist of the N-terminal region with 0, 1, 2 inserts (0N, 1N, and 2N), prolin-rich domain (PRD), microtubule binding domain with three or four repeats (3R or 4R), and C-terminal region. Intraneuronal accumulation of filamentous tau protein is a hallmark of a wide range of neurodegenerative diseases such as AD and frontotemporal dementia, collectively termed tauopathy (Ballatore et al., 2007; Goedert et al., 2017). The filamentous tau aggregates in different tauopathy patients were shown to have different composition. For example, all six isoforms were observed in AD patients, while only 4R-or 3R-isoform of tau was detected exclusively in progressive supranuclear palsy (PSP) and Pick’s disease (PiD), respectively (Goedert et al., 2017).

Recently, near-atomic resolution structures of tau filaments were determined by cryo-EM (Fitzpatrick et al., 2017; Falcon et al., 2018; Zhang et al., 2019). Figure 2F shows high-resolution structure of 2N4R tau filaments derived from an AD brain tissue (Fitzpatrick et al., 2017). Residues 306–378 form a core of the filament which adopts the cross-β fold with β-helix structures, while the rest of the residues are disordered and form the fuzzy coat. The two protofilaments form twisted filaments with interfacial region of residues 332–336. Tau filaments (2N4R) induced by the addition of heparin were shown to have at least four distinct molecular structures (Figures 2G–J), which are quite different from that in AD (Figure 2F; Zhang et al., 2019). In addition, tau filaments (2N3R) from patients with PiD have a distinct cross-β fold consisting of residues 254–378 (Figure 2K; Falcon et al., 2018), highlighting structural diversities of tau filaments. The distinct molecular conformations of tau filaments may have different biological activities associated with different disease phenotypes. More detailed investigation of biological activities including toxicity and self-propagation properties of diverse tau filaments will lead to a deeper understanding of the phenotype diversities of tauopathies. Finally, it is worth emphasizing that the heparin-induced *in vitro* tau filaments (Figures 2G-J) adopt completely different structures from those of *in vivo* tau filaments (Figures 2F,K), highlighting the importance of using physiological conditions for the biophysical studies of misfolded protein aggregates. This also raises an important question whether recombinant aggregation-proneproteins including tau and α-synuclein can form disease-associated filamentous aggregates observed *in vivo*, and thus special care must be taken when developing therapeutic intervention on the basis of *in vitro* filament structures.

## Self-Propagation of Misfolded Strains in a Prion-Like Manner

Misfolded protein aggregates are initially found in a distinct brain region depending on the type of diseases. At a later stage, the protein aggregates gradually spread in a predictable manner (Frost and Diamond, 2010; Kim and Holtzman, 2010; Lee et al., 2010; Westermark and Westermark, 2010; Goedert et al., 2017). For example, tau aggregates are initially found in the locus coeruleus, entorhinal cortex and hippocampal formation, which are associated with the memory, in Alzheimer’s patients. Over time, the misfolded tau aggregates begin to appear in the neighboring regions of the temporal and frontal lobes, which affect cognitive functions such as learning and speech. In addition, the intracerebral injection of brain extracts containing misfolded protein aggregates into transgenic mice induced the formation of misfolded tau aggregates at near injection site (Clavaguera et al., 2009, 2013; Iba et al., 2013). The induced aggregates propagate systematically from the injection site and spread to distant regions, promoting disease phenotypes similar to those of the corresponding human diseases (Meyer-Luehmann et al., 2006). It was also demonstrated that misfolded tau aggregates can be taken by neurons in cell culture and the tau aggregates were able to stimulate tau aggregation inside the neurons. In addition, recent studies revealed that tau aggregates propagate from neuron to neuron, suggesting that the misfolded aggregates spread through neuronal networks (Gibbons et al., 2019).

The prion-like behavior was also observed in other misfolded proteins such as Aβ and α-synuclein. Intracellular injection of brain extracts containing misfolded α-synuclein aggregates into transgenic mice induced motor dysfunction similar to those of PD (Sacino et al., 2014). In addition, misfolded aggregates derived by purified recombinant α-synuclein were shown to be able to induce α-synuclein aggregation *in vivo* (Peng et al., 2018). Injection of the brain extracts including Aβ plaques as well as synthetic Aβ aggregates seeded Aβ deposition in mice (Meyer-Luehmann et al., 2006). These experimental evidences clearly suggest that the aggregation-prone pathological proteins have the ability of propagating their pathological conformations like a prion. Elucidation of the molecular mechanism of the self-propagation is critical to developing therapeutic agents that can inhibit the propagation process.

During the self-propagation process, the misfolded protein aggregates recruit soluble monomeric proteins and induce conformational changes to misfolded conformers. The end of the filamentous aggregates may interact with the soluble proteins or surface of the aggregates may also recruit the monomeric proteins, promoting intermolecular associations of the monomeric proteins. Distinct strains with different molecular structure and morphologies may interact with monomeric proteins in different ways to induce distinct conformational changes to propagate their unique molecular conformations. Elucidation of the molecular basis of the interactions between the misfolded strains and monomeric proteins would be of great importance in developing therapeutic strategies to block the self-propagation process. Recently solved high-resolution structures of the misfolded filamentous aggregates will be of great use in investigating interactions between the filaments and monomeric proteins.

## Cross-Talk Between Misfolded Proteins

Traditionally, misfolding and aggregation of a single protein is thought to be implicated in each neurodegenerative disorder with an independent pathological process. There is, however, an increasing body of evidence that suggests multiple neurodegenerative diseases are significantly overlapped. First, co-deposition of α-synuclein and tau was observed in ADRD (Clinton et al., 2010; Irwin et al., 2013; Moussaud et al., 2014; Nonaka et al., 2018). For example, misfolded α-synuclein aggregates were found in more than 50% of AD, Down’s syndrome, and familial AD cases (Lippa et al., 1998, 1999; Hamilton, 2000). Tau aggregates were also detected in patients with Parkinson’s disease dementia and the amount of tau aggregates is correlated well with cognitive decline (Forman et al., 2002; Irwin et al., 2013). Second, it was previously shown that αsynuclein and tau can synergistically promote their mutual aggregation, suggesting that synergistic interactions between the two pathological proteins might exacerbate ADRD pathology (Giasson et al., 2003; Oikawa et al., 2016). In particular, misfolded α-synuclein aggregates with distinct molecular conformations (strains) were shown to differentially induce the formation of distinct tau strains *in vivo* (cross-seeding; Guo et al., 2013). These results indicate that AD and PD pathologies are significantly overlapped presumably due to synergistic interactions between tau and α-synuclein, highlighting the complexity of ADRD pathogenesis.

Recent studies have also shown that patients with type 2 diabetes have a higher risk for AD. In addition, about 80% of Alzheimer’s patients develop type 2 diabetes, suggesting that AD might be linked to type-2 diabetes (Jucker and Walker, 2011). Misfolding and aggregation of a small 37-residue peptide (islet amyloid polypeptide, IAPP) is associated with type-2 diabetes. Misfolded IAPP aggregates were also shown to be able to seed Aβ aggregation *in vitro* as well as *in vivo* (Hu et al., 2015; Moreno-Gonzalez et al., 2017). Intracellular injection of pancreas IAPP aggregates into transgenic mice induced higher extracellular Aβ deposition in brain, resulting in more severe learning and memory deficits (Moreno-Gonzalez et al., 2017). These experimental results indicate the presence of synergistic interactions between the two aggregation-prone proteins, aggravating the diseases.

Despite the growing evidence of the cross-talk among protein misfolding disorders, molecular mechanism of the synergistic interactions remains to be determined. Aggregation-prone proteins may interact directly with each other to promote mutual aggregations, as was demonstrated *in vitro*. It is, however, unclear how the filamentous aggregates mutually promote aggregation of the other aggregation-prone proteins. Even though the misfolded filamentous aggregates of the three proteins are stabilized by similar interactions such as hydrogen bonding, salt-bridge, and hydrophobic interactions, they adopt completely different cross-b folds as shown in Figures 1B, 2. These different structures suggest that disordered regions rather than the amyloid core may interact with the monomers of different aggregation-prone proteins, accelerating aggregation. For example, negatively charged C-terminal region (101–140; pI, 3.4) that is not involved in α-synuclein filament core may disrupt long-range interactions present in positively charged tau (pI, 11.4; Mukrasch et al., 2009), destabilizing tau monomers and subsequently promoting aggregation, and vice-versa. Determination of high-resolution structures of filamentous aggregates cross-seeded by other misfolded aggregates will be required to better understand the cross-seeding activities. Misfolded protein aggregates may also indirectly induce aggregation of the other pathological proteins by interfering protein quality control systems, increasing cellular vulnerability to misfolding and aggregation.

## Concluding Remarks

It is increasingly evident that aggregation-prone proteins behave like a prion that adopts diverse molecular conformations with self-propagation properties and cross-seeding activities. Recent advances in cryo-EM and solid-state NMR techniques allowed high-resolution structures of misfolded filamentous aggregates to be determined at near-atomic resolutions, which revealed diverse molecular conformations. Additional structural studies of filamentous aggregates cross-seeded by another misfolded proteins and *in vivo* filaments extracted from patient’s brains will greatly enhance our understanding of molecular basis for the diverse molecular conformations. Investigation of their biological activities such as toxicity and cross-seeding ability will also provide more detailed insights into structure-function relationship of the conformationally diverse misfolded strains. Finally, it is important to note that the high-resolution structures described in this review were determined only for highly ordered filamentous protein aggregates. Oligomeric intermediate states that are believed to be real cytotoxic species may have different molecular structures. Structural studies of the oligomers using cryo-EM and solid-state NMR are, however, of great challenge due to their heterogeneous, transient nature. A recent study showed that oligomeric species dissociated from filamentous aggregates exhibited cytotoxic activities (Ghag et al., 2018), and thus high-resolution structures of the amyloid filaments described above may help investigate structural features of the cytotoxic oligomers.

## Author Contributions

KL wrote the manuscript.

## Conflict of Interest Statement

The author declares that the research was conducted in the absence of any commercial or financial relationships that could be construed as a potential conflict of interest.
